# Accessible data curation and analytics for international-scale citizen science datasets

**DOI:** 10.1038/s41597-021-01071-x

**Published:** 2021-11-22

**Authors:** Benjamin Murray, Eric Kerfoot, Liyuan Chen, Jie Deng, Mark S. Graham, Carole H. Sudre, Erika Molteni, Liane S. Canas, Michela Antonelli, Kerstin Klaser, Alessia Visconti, Alexander Hammers, Andrew T. Chan, Paul W. Franks, Richard Davies, Jonathan Wolf, Tim D. Spector, Claire J. Steves, Marc Modat, Sebastien Ourselin

**Affiliations:** 1 King’s College London, School of Biomedical Engineering & Imaging Sciences, London, SE1 7EU United Kingdom; 2 University College London, MRC Unit for Lifelong Health and Ageing, Department of Population Health Sciences, London, WC1E 7HB United Kingdom; 3 University College London, Centre for Medical Image Computing, London, WC1E 6BT United Kingdom; 4 King’s College London, Department of Twin Research and Genetic Epidemiology, Westminster Bridge Road, London, SE1 7EH United Kingdom; 5 Massachusetts General Hospital, 55 Fruit Street, GRJ 825C, Boston, MA 02116 United States; 6 Lund University, Diabetes Centre, CRC, SUS Malmö, Jan Waldenströms gata 35, House 91:12, SE-214 28 Malmö, Sweden; 7 Zoe Limited, 164 Westminster Bridge Road, London, SE1 7RW United Kingdom

**Keywords:** Epidemiology, Research data

## Abstract

The Covid Symptom Study, a smartphone-based surveillance study on COVID-19 symptoms in the population, is an exemplar of big data citizen science. As of May 23rd, 2021, over 5 million participants have collectively logged over 360 million self-assessment reports since its introduction in March 2020. The success of the Covid Symptom Study creates significant technical challenges around effective data curation. The primary issue is scale. The size of the dataset means that it can no longer be readily processed using standard Python-based data analytics software such as Pandas on commodity hardware. Alternative technologies exist but carry a higher technical complexity and are less accessible to many researchers. We present ExeTera, a Python-based open source software package designed to provide Pandas-like data analytics on datasets that approach terabyte scales. We present its design and capabilities, and show how it is a critical component of a data curation pipeline that enables reproducible research across an international research group for the Covid Symptom Study.

## Introduction

Mobile applications have enabled citizen science^1–4^ projects that can collect data from millions of individuals. The Covid Symptom Study^5^, a smartphone-based surveillance study on self-reported COVID-19 symptoms started in March 2020, is an exemplar of citizen science. As of May 23rd, 2021, the study contains over 360 million self-assessments collected from more than 5 million individuals. The data is provided as daily CSV (comma separated value) snapshots that are made available to both academic and non-academic researchers to facilitate COVID-19 research by the wider community.

The Covid Symptom Study dataset presents some demanding data curation challenges. We define data curation as involving, but not being limited to, the application of a set of transformations to the raw data. Such transformations include generation or application of metadata, cleaning of noisy and inconsistent values or relationships between values, and generation of consistent derived measures more suitable for further analysis. Erroneous values, changing schemas and multiple contemporary mobile app versions all add complexity to the task of cleaning and consolidating datasets for downstream analysis. Data curation must be performed effectively as a precondition for reproducible science. In terms of the data curation definition provided by Lee *et al*.^6^, we focus primarily on’managing and sharing data’ as defined in Table 1 of their publication.

**Table 1:** Time taken to import the Patient table from the Covid Symptom Study 2021/05/23 snapshot.

Table	Time to import patent table (seconds)			
	ExeTera	Pandas	Dask	PostgreSQL
Patients	**84.81**	(141.01)	*Memory*	105.88
Assessments	2266.65	*Memory*	*Memory*	**1946.60**
Tests	74.04	**37.46**	434.40	42.37

Figures in parentheses denote that the import required more than 32 GB of memory to succeed. *Memory* denotes that the import was unable to succeed as it required more than 256 GB of memory. Figures in **bold** indicate the best import time.

The primary challenge in curating and analysing Covid Symptom Study data is scale. Scale adds complexity to otherwise simple operations. Python-based scientific computing libraries such as Numpy^7,8^ and Pandas^9^ are ubiquitous in the academic community, but they are not designed to scale to datasets larger than the amount of RAM (random access memory) on a given machine. Commodity hardware in 2021 is typically equipped with 16 to 32 GB (gigabytes) of memory. Larger amounts of RAM necessitate expensive server-grade hardware, and doubling memory only doubles the size of dataset that can be handled.

Datasets too large for standard Python scientific computing tools can be moved to datastores, either traditional, relational SQL databases or distributed NoSQL datastores, such as key-value stores. Each type of datastore comes with its own design philosophy and performance trade-offs^10^. Such datastores can operate on datasets far larger than RAM but involve considerable additional complexity^11^ through installation and maintenance burdens, and the need to learn new APIs and concepts. Additional computing power can be accessed through cloud computing but this brings ongoing costs, particularly for high-memory compute instances. Cloud computing also adds complexity to a solution, as cloud APIs are non-trivial to work with.

Although most of the principal Python libraries for data science are not designed to work with larger-than-RAM datasets (see https://pandas.pydata.org/pandas-docs/stable/user_guide/scale.html), they provide a rich set of design choices and concepts that have proven successful with and are well understood by data scientists within the Python-using scientific community. By building on those design choices and concepts, but focusing on the provision of key implementation choices and algorithms that are critical for scaling beyond RAM, one can create highly scalable data analysis software with an API familiar to users of the Python ecosystem.

Software packages with more scalable implementations of Pandas DataFrames exist. Dask (see https://dask.org) and PySpark^12^, for example, offer powerful graph-based execution models capable of performing very large calculations over multiple cores and machines. They also provide dataframe-equivalent implementations which appear promising. As we will demonstrate for Dask, the inability to cherry-pick columns from the data frame causes fundamental issues when dealing with billion element fields, however. Vaex^13^ is another alternative that provides similar functionalities at scale, but it is not fully open source as some components are enterprise access only.

Scale is not the only challenge to consider. Reproducibility of analyses, especially across large research teams, is also critically important. When data cleaning and generation of analytics is done in an ad-hoc fashion, it is easy to generate subtly different derivations of a given measure, causing inconsistencies across research teams and research outputs. Furthermore, full reproducibility requires algorithms to be treated as immutable, so that the application of a particular algorithm to a particular snapshot of data guarantees identical results, hardware notwithstanding.

The Covid Symptom Study is delivered as a series of timestamped snapshots. Corresponding entries can be modified between snapshots, and unless the dataset explicitly records changes to entries over time, one can only determine the difference by comparing the snapshots, which exacerbates scaling issues. Updating an analysis from one snapshot to another without such comparison compromises the interpretability of the updated results in a way that is less obvious when working with individual snapshots.

To address the challenges described above, we have created ExeTera, a software that enables sophisticated analysis of tabular datasets approaching terabyte scale, such as the Covid Symptom Study dataset, on commodity hardware. ExeTera has an API designed to be familiar to users of Pandas, a ubiquitous tabular data analysis library within the Python ecosystem, allowing researchers to use their existing expertise. Pandas’ design is itself based on the data frame of R (see https://www.r-project.org/), another data analysis software popular with the scientific community.

ExeTera can analyse datasets significantly beyond RAM scale by paying attention to two factors. The first is a careful selection of data representation that provides the ability to perform operations on tables without them ever being fully resident in memory. The second is the observation that certain operations become trivially scalable when the data is presented in lexical order. With an appropriate representation and the ability to sort table row order, commodity hardware with modest amounts of RAM can perform sophisticated analyses on datasets approaching or even exceeding terabyte scales.

Although we present ExeTera in the context of the Covid Symptom Study, it is designed to be applicable to any dataset of related, tabular data. For the Covid Symptom Study, we have created ExeTeraCovid, a repository of scripts and notebooks that uses ExeTera to create reproducible end-to-end data curation workflows.

The data curation workflow for the Covid Symptom Study has three stages. The first stage is a transformation of the data from text-based CSV to concrete data types where each row is parsed for validity given metadata describing the data types. The second stage is the application of a standardised set of cleaning and imputation algorithms that remove duplicate rows, detect and recover problematic values, and generate derived values from the source data. The third stage is the development and subsequent publishing of end-to-end scripts that, given a daily snapshot, can replicate a given analysis.

In the following sections, we present ExeTera’s underlying capabilities and design, as well as its usage within the Covid Symptom Study as a case study.

## Results

ExeTera’s performance has been benchmarked against a combination of artificial data and Covid Symptom Study data. We examine performance and scalability of ExeTera and its alternatives for key operations such as importing data, reading subsets of data, and performing joins between tables. We also demonstrate Exetera’s ability to generate a journalled dataset from snapshots allowing longitudinal analysis able to account for destructive changes between corresponding rows of the different snapshots. Finally, we present an example of ExeTera’s analytics capabilities.

We have selected Pandas, PostgreSQL, and Dask to benchmark against ExeTera. Pandas has been selected as the baseline against which we are testing ExeTera. Dask has been selected as it is the most popular Python-based open-source library with a Pandas-like API that is explicitly designed for distribution and scale. PostgreSQL has been selected as it is the most popular fully open-source relational database and is widely used in academia.

Performance is measured on an AMD Ryzen Threadripper 3960 × 24-core processor with 256 GB of memory. All key benchmarking processes are limited to 32 GB of memory where technically feasible although the construction of datasets for testing is allowed to use more than 32 GB. The data is read from and written to a 1 TB (terabyte) Corsair Force MP600, M.2 (2280) PCIe 4.0 NVMe SSD.

### Datasets

Benchmarking is performed on part of the Covid Symptom Study dataset and on synthetic data designed to mimic the relationship between the Patient and Assessment tables of the Covid Symptom Study dataset.

#### Covid Symptom Study

We use the Covid Symptom Study data snapshot from the 23rd of May 2021, unless otherwise stated. We make use of the three largest tables:

- Patients: This table has 202 columns and 5,081,709 rows. It contains biometrics, location, long term health status and other such values that rarely change over time.
- Assessments: This table has 68 columns and 361,190,557 rows. It includes current health status and symptoms, behavioural habits such as exposure to others and mask wearing, and other factors that are logged on an ongoing basis by contributors.
- Tests: This table has 20 columns and 6.979,801 rows. Tests are logged whenever a patient gets a Covid test and updated with the result of the test when available. Test parameters such as the test type and date are also recorded here.

#### Artificial data

In order to demonstrate the ability for ExeTera to scale relative to technologies that explicitly handle larger than RAM datasets, we construct simple artificial tables with increasingly large column counts. This is used to evaluate joins at row-counts beyond those of the Covid Symptom Study. The code to generate these tables is part of the ExeTeraEval repository, listed in the *Code Availability* section.

The artificial dataset has two tables. The left table is an artificial analogue of the patient data from the Covid Symptom Study. The right table is an artificial analogue of the assessment data from the Covid Symptom Study. Each patient has 0 or more entries in the assessment table, with a mean of 10 entries per patient.

### Import performance

We measure the performance of the import activities that must be carried out in order to perform analysis on the data. We define ‘import’ to mean anything that must be done to the data representation to minimise/eliminate the parsing required upon subsequent loads.

For ExeTera, we use the exetera import operation that converts CSV data to ExeTera’s HDF5-based datastore format.

For Pandas, reading from CSV imposes an expensive parse step every time a dataframe is loaded from drive, so we perform a single preliminary load from CSV as an import, assigning the appropriate metadata so that the columns are strongly typed, and then save the data as a HDF5 file. All subsequent operations are then benchmarked on the HDF5-based dataframe.

For Dask, we perform a similar operation in which we read from CSV, assigning metadata and writing to partitioned HDF5 files. Note that Dask uses Pandas DataFrames for its partitions. As with Pandas, all subsequent operations are then benchmarked on the HDF5 data.

For PostgreSQL, we import the data through the execution of a SQL script that creates the tables and then reads the data from CSV. We include the cost of setting up primary keys and foreign key constraints used to optimise subsequent operations. In the case of the Covid Symptom Study Patient data, this involves the removal of duplicate rows that are present in the Patient table. This allows the primary key constraint to be established on the id column and the foreign key constraint to be established on patient_id column for the Assessment and Test tables.

The results are shown in Table 1. Where the process was not able to complete using 32 GB of memory, the result is shown in brackets. ‘Memory’ denotes that process was not able to complete using 256 GB.

It should be noted that the presence of columns containing long strings of natural language significantly impact both Pandas’ and Dask’s ability to convert the Patient table from CSV to HDF5. A detailed analysis of why is presented in the *Avoiding scaling issues with long string data* section.

### Reading columns

Reading individual fields from datasets is the most common operation performed during data analysis. For a typical analysis, we might only need a small subset of the columns in the dataset, and so it is highly beneficial if the cost of reading this data is proportional to the number of columns being loaded. This test is carried out on the Patient table and the results are shown in Table 2.

**Table 2:** Time taken taken to perform reads of a number of columns of the Patient table from the Covid Symptom Study 2021/05/23 snapshot.

Field count	Time to read patient fields (seconds)			
	ExeTera	Pandas	Dask	PostgreSQL
2 fields	**0.068**	(142.56)	*NA*	3.22
4 fields	**0.075**	(143.21)	*NA*	8.24
8 fields	**0.084**	(142.35)	*NA*	9.71

Figures in parentheses denote that the read required more than 32 GB of memory to succeed. *NA* denotes that the operation could not be performed due to the dataset not being successfully imported. Figures in **bold** indicate the best read time.

### Join performance

**Fig. 1:**
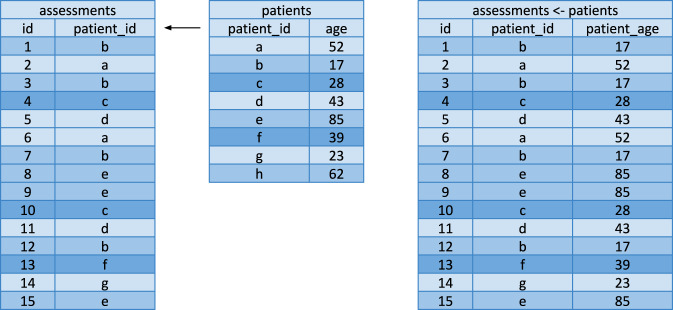
A left join of a simplified dummy Patent and Assessment dataset. This left join matches patient_id entries in the Patient table to patient_id in the assessment table. The appropriate patient_age values are mapped to the corresponding rows from the Assessment table.

Here we measure the performance of join operations on the dataset. We measure this both with a snapshot of the Covid Symptom Study and with a synthetic dataset for different row lengths. Figure 1 illustrates a left join operation.

We perform the following joins on the Covid Symptom Study, shown in Table 3:

- Left join: Patient < - Assessment
- Left join: Assessment < - Patient
- Left join: Patient < - Test
- Left join: Test < - Patient

**Table 3:** Time taken to perform left joins on the Covid Symptom Study 2021/05/23 snapshot.

Tables	Covid Symptom Study left join time (seconds)			
	ExeTera	Pandas	Dask	PostgreSQL
Assessments < - Patients	**224.13**	*NA*	*NA*	391.09
Patients < - Assessments	**224.31**	*NA*	*NA*	394.33
Tests < - Patients	**7.62**	*NA*	*NA*	9.92
Patients < - Tests	**7.97**	(295.08)	*NA*	10.51

Figures in parentheses denote that the join required more than 32 GB of memory to succeed. *NA* denotes that the operation could not be performed due to the dataset not being successfully imported. Figures in **bold** indicate the best join time.

The results for left joins performed on artificial data are shown in Table 4.

**Table 4:** Time taken to perform left joins on an artificial dataset.

Right row count	Artificial left join time (seconds)			
	ExeTera	Pandas	Dask	PostgreSQL
1,000,000	0.594	**0.174**	0.488	0.697
2,000,000	0.662	**0.278**	1.22	1.47
3,000,000	0.744	**0.362**	1.76	2.22
4,000,000	0.805	**0.450**	2.11	2.89
6,000,000	0.946	**0.675**	3.02	4.51
8,000,000	1.09	**0.872**	4.29	6.05
10,000,000	1.21	**1.06**	5.28	7.40
20,000,000	**1.88**	2.09	12.71	14.41
30,000,000	**2.54**	3.24	20.92	21.81
40,000,000	**3.25**	4.36	30.62	32.61
60,000,000	**4.55**	6.56	54.62	48.09
80,000,000	**5.88**	9.19	*Failed*	70.83
100,000,000	**7.01**	11.93	113.76	87.04
200,000,000	**13.60**	24.66	*Failed*	198.87
300,000,000	**19.68**	*Memory*	*Failed*	280.83
400,000,000	**27.52**	*Memory*	*Failed*	379.4
600,000,000	**39.86**	*Memory*	*Failed*	578.19
800,000,000	**53.74**	*Memory*	*Failed*	767.43
1,000,000,000	**69.19**	*Memory*	*Failed*	964.67

Row counts shown are for the right table, which has 10x the row count of the left table (e.g. 100,000,000 rows in the left table when the right table has 1,000,000,000 rows). *Memory* denotes that the import was unable to succeed as it required more than 32 GB of memory. *Failed* denotes that the operation was unable to complete due to reasons other than memory. Figures in **bold** indicate the best import time.

ExeTera joins are performed through the exetera.core.dataframe.merge function. In order to access highest-scale joins, the keys must be in lexical order. The dataframe.merge function requires a destination ExeTera DataFrame instance to write to, so the times presented measure the time taken to read the source dataframes, perform the merge and write to the destination dataframe.

Joins in Pandas are carried out through the pandas.merge function. Like ExeTera, Pandas takes advantage of lexically ordered keys to improve merge performance, and so we measure merges on Pandas with ordered keys.

Dask joins are performed through dask.dataframe.merge. As with ExeTera, the merge results must be written to a destination dataframe serialised to disk to achieve scale, so this is part of the measurement.

PostgreSQL joins are performed through the JOIN statement in SQL. As we are concerned with measuring scale, we write the results of the JOIN to a table using CREATE TABLE x as followed by the SELECT and JOIN statements.

To understand the ultimate join scale limitations of the different technologies, we do not restrict the memory usage of the dataset generation process, but the process that performs the join is restricted to 32 GB of memory, except for Dask, for which we did not determine a way to limit the working set of the distributed processes.

### Journalling operation

ExeTera can combine snapshots of datasets to create a journalled dataset, keeping multiple, timestamped copies of otherwise destructive changes to corresponding records between the snapshots. Table 5 shows the results of journalling together shapshots of the Covid Symptom Study from the August 1st 2020 and September 1st 2020, and the time taken to do so.

**Table 5:** Journalling Covid Symptom Study snapshots from 1st August 2020 and 1st September 2020.

Data source	Journalling dataset snapshots			
	Patients	Assessments	Tests	Diet
August 1st row count	4,402,930	129,423,329	749,937	659
September 1st row count	4,480,270	153,655,115	991,128	1,291,237
Rows only in old	2,519	108,231	485	0
Rows only in new	86,301	243,400,017	241,676	1,290,578
Rows updated	1,632,849	702	18,169	630
Rows not updated	2,761,120	129,314,396	731,283	29
Journalled row count	6,122,080	153,764,048	1,009,782	1,291,867
Time to import (seconds)	145.1	2273	6.616	8.179

This table shows results in terms of row counts and the time taken to perform the journalling.

### Analytics

**Fig. 2:**
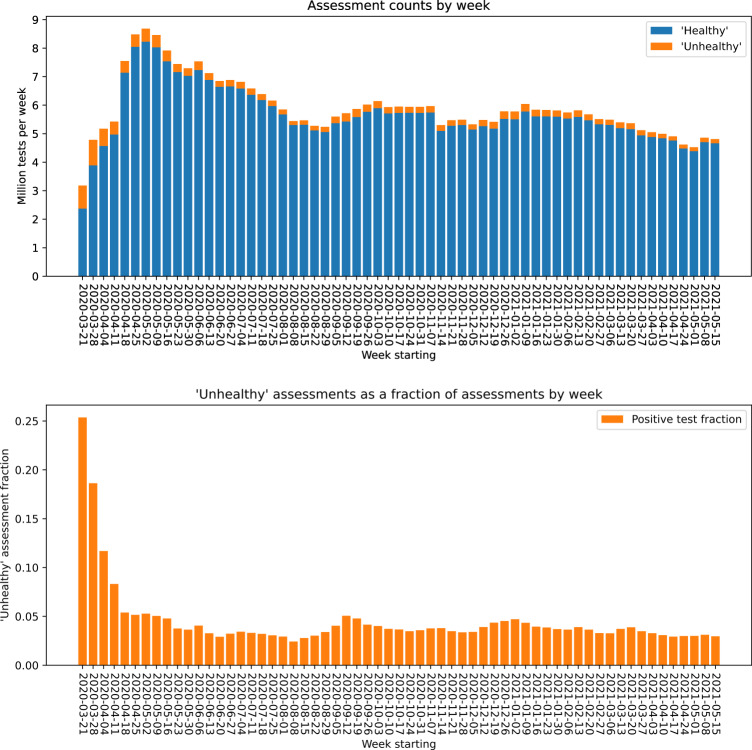
Seven day summary of assessments from the Covid Symptom Study snapshot dated 16th May 2021. The upper chart shows the number of assessments, coloured by whether the patient logged as healthy or unhealthy. The lower chart shows the assessments logged as unhealthy as a fraction of assessments logged for that seven day period.

ExeTera provides the ability to load data very efficiently, as seen in Table 2. Once loaded, analytics can be performed through use of libraries such as Numpy and Matplotlib^14^, using tools that researchers are familiar with, such as Jupyter Notebook^15^. Figure 2 shows a histogram of healthy and unhealthy assessment logs bucketed into seven day periods that must parse 361 million assessments to generate its results.

## Discussion

We have presented ExeTera, a Python software package that enables data analytics on datasets of related tables approaching terabyte scales. ExeTera demonstrates that a commodity computer is capable of performing analyses at scale, given appropriate software. It is a low-complexity solution from a user standpoint, designed to be familiar to data scientists who are familiar with Python’s scientific computing ecosystem, and does not require the user to be aware of concurrency or data partitioning factors.

ExeTera’s primary novelty is a design and implementation that allows it to carry out highly scalable operations between dataframes. Its join functionality, in particular, scales as well as PostgreSQL and even outperforms it in benchmarks on both synthetic and Covid Symptom Study data. ExeTera is written in Python, requires no server and is trivially installable through the Python software package manager Pip (https://packaging.python.org/key_projects/#pip).

ExeTera’s ability to scale is provided through streamable implementations of key operations operating on a columnar data format discussed in detail in the Methods section. By taking advantage of the fact that large tabular datasets such as the Covid Symptom Study typically have orders of magnitude more rows than columns, ExeTera can achieve excellent scaling and performance.

That said, ExeTera currently has two practical scale limitations. Firstly, although key operations scale to drive size, some of the more mundane operations do not yet have streaming implementations. As a result, the user must maintain some awareness of scaling factors when manipulating large numbers of fields concurrently. Secondly, ExeTera is single core at present.

We plan to resolve these limitations by adopting graph scheduling and processing technology capable of managing array operations through dependency management and task scheduling. As of the time of writing, we are evaluating Dask for this purpose. Dask has powerful task decomposition and scheduling capabilities that provide distributed, scalable array operation primitives. Rather than making use of Dask’s DataFrame, we will build our own distributed, scaled versions of key algorithms on the Dask array API as we do currently with Numpy arrays. This addresses both limitations; all operations on ExeTera fields become streaming by default if they are based on Dask arrays rather than Numpy arrays and the scheduler provides multi-core / multi-node distribution that effectively eliminates the need for the user to concern themselves with memory usage. Dask is also integrated with more specialised back-ends such as Nvidia’s RAPIDs^16^, enabling execution of distributed graph processing across GPU clusters. Dask integration should allow ExeTera to handle datasets well into the multi-terabyte range with minimal overhead.

ExeTera’s ability to store multiple snapshots in a journalled format enables researchers to perform full longitudinal analysis on otherwise unjournalled datasets and facilitates the ability to move between snapshots while being able to properly explore the impact of doing so on analyses.

ExeTera has played a critical role in supporting the Covid Symptom Study research effort and enables the ExeTeraCovid repository, which implements the data curation and analytics scripts for many of the papers published as part of the joint research effort between Zoe Ltd., King’s College London, Massachusetts General Hospital, and Lund University, a selection of which are referenced here^17–24^.

Reproducibility is enabled primarily through convention at present. The ExeTeraCovid project is built on top of ExeTera and achieves reproducibility through the convention that algorithms are treated as immutable once implemented and deployed. This means that any future version of ExeTeraCovid will have any versions of algorithms that are implemented now as well as future refinements to those algorithms. This allows the effect of changing an algorithm to easily be quantified and allows users to update between ExeTeraCovid releases without affecting analyses. Reproducibility will be directly supported in a future version of ExeTera.

ExeTera is still at an early stage of development but has a funded, dedicated development team. ExeTera’s roadmap addresses the advancement of its fundamental performance and capabilities, the breadth of its analytics API, and the richness of its data curation feature set, and is available on the respository wiki listed in the *Code Availablity* section.

## Methods

This section is split up into three parts. The first two deal with ExeTera and its design and implementation. The third part deals with ExeTeraCovid and our data curation methodology for the Covid Symptom Study data.

### ExeTera design

ExeTera achieves its performance through careful design and implementation decisions. We detail the most important of these here with the background context that motivates them.

#### Domains of scale

In order to successfully perform analyses on large datasets such as the Covid Symptom Study, it is necessary to be able to handle data tables that cannot fit into RAM. Data size and structure, and the set of operations needed to handle the dataset, must be addressed. We can define three scale domains that necessitate a change of approach at their boundaries.

##### RAM Scale (1 GB to 16 GB)

This is the scale at which the dataset entirely fits in the computer’s RAM. Commodity laptops and desktops used by researchers typically have between 16 and 32 GB of RAM. Loading the data can inflate its memory footprint depending on the datatypes used, and operations can multiply memory requirements by a small constant factor, but provided peak memory usage does not dramatically exceed RAM, researchers can make use of programming languages with numerical/scientific libraries such as Numpy or Pandas to effectively analyse the data.

##### Drive Scale (16 GB to 1 TB)

At drive scale, only a portion of the dataset can fit into RAM at a given time, so specific solutions are required to effectively stream the dataset from drive to memory. Datastores become a more compelling option at this scale, as they already have memory efficient, streaming versions of the operations that they support, but their usage may not be desirable due to the need to learn a new language or API, and the installation and maintenance burden they represent. This is the scale of dataset that ExeTera currently targets.

##### Distributed Scale (>1 TB)

At distributed scale, the use of server-based datastores is typically mandatory. It becomes necessary to redesign operations to exploit distributed computing across many nodes. Selection of appropriate datastore technology becomes critical, with specific datastore technologies addressing different roles within the overall system. This scale will be targeted by ExeTera in future development through the incorporation of Dask or similar graph scheduling and processing technology.

#### Serialised data representation

In order to handle datasets that are larger than memory the data must be stored on a drive and only a subset of the data loaded into RAM at any given time. Picking an appropriate serialized data representation is a key factor in achieving fast, scalable operations at any scale. Text-based formats such as CSV are commonly used to portably represent large datasets, but they come with many drawbacks, primarily a lack of strong typing/metadata and an inability to rapidly index to a given location in the dataset. These issues become severe at scale, and so an alternative serialised data representation is required. Binary, strongly typed data formats that can be copied from their storage representation directly to memory and vice-versa are optimal for this purpose. Assuming appropriate binary formats are used, the key question is to know how to organise the data, and this is where ExeTera differs from the software packages against which it has been benchmarked.

##### Data representation: row-local vs. column-local data layouts

**Fig. 3:**
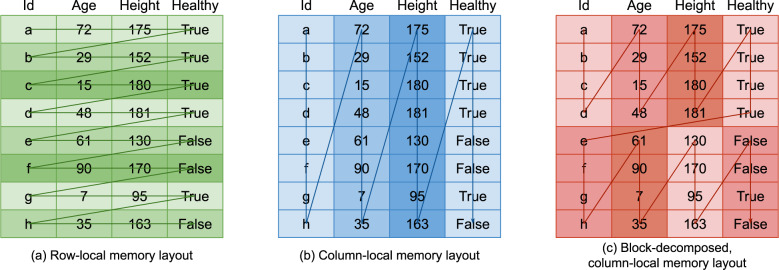
Figure illustrating different memory layout strategies. In each memory layout, the arrow indicates the ordering of the cells in memory relative to each other.

Data storage formats can be classified as primarily row-local or primarily column-local in terms of their data layouts (see Fig. 3). Row-local data layouts store groups of related columns for a given data entry together in memory. Column-local data layouts store all data entries for a specific column together in memory. Alternatively, the two approaches can be combined to create block-decomposed data layouts. The selection of an appropriate data layout is a critical factor in the potential performance of operations on the data (see https://people.freebsd.org/ lstewart/articles/cpumemory.pdf.

##### Row-local data layout

Row local data layout allows multiple columns for a given record to be updated very efficiently as the required values for the row are located together in memory. It is less suitable for operations involving entire columns, as this involves the reading of many disjoint memory locations to access the column contents as they are spread over the table representation.

CSV files are almost always interpreted in a row-local fashion. Each entry for each column is delimited by a separator, typically a comma. Rows are terminated by an unescaped newline character. CSV cells can contain separator and newline characters, and so additionally require escape characters (typically double quote) that indicate cells containing separator / newline characters. Additionally, such escaped cells can also contain escaped characters.

SQL databases also store their data in a primarily row-local format, although they typically make some use of blocking^10,25^. This allows them to be fast when used for workloads in which many point updates (often several fields within a single row) are being made concurrently, but it means that monolithic reads of entire columns require consolidation and copying of many small regions of serialised memory rather than a single monolithic read. SQL databases have highly refined algorithms that are able to maximise performance given the underlying data representation.

##### Column-local data layout

Column-local data storage enables very efficient access to a given column. All the entries for a given column are (effectively) contiguous in memory and loading a single column can be done in an optimal fashion by the memory subsystem. When a dataset has many columns and a given operation operates on a smaller number of those columns, scaling the operation is a far simpler proposition. Conversely, column-local data storage performance suffers when multiple small updates are being made to multiple large columns. Reading and updating a row across multiple columns involves a series of disjointed reads in this case. This is the approach taken by ExeTera.

##### Block-decomposed layouts

Block-decomposed formats attempt to amortise the cost of the poorly performing operation at the expense of the strongly performing operation. In the case of block-decomposed column-local formats, such as that employed by Pandas, they store blocks of columns of the same datatype together in memory as 2D Numpy arrays (see https://github.com/pydata/pandas-design/blob/master/source/internal-architecture.rst).

##### ExeTera is pure column-local

ExeTera uses a purely column-local data orientation with no block decomposition. We consider the pure column-local approach is critical to scalability for several reasons. Firstly, it is the lowest complexity solution from a code standpoint. If a block-decomposition strategy is used, code paths must account for operations that work across different blocks as well as operations that ideally want to optimise working within a single block. This necessarily involves more code and additional complexity. Secondly, our goal is to maximise the row-counts that we can effectively handle for operations that have not yet been provided streaming implementations. By being able to load a single column into memory at a time, non-streaming operations can be performed on columns containing billions of rows for most datatypes. Thirdly, such columns can be loaded with single contiguous reads and we can extend ExeTera to use memory mapping (a fast way of mapping a part of a drive directly into RAM) very simply. Finally, each column being stored separately allows us to move to compression techniques such as adaptive run-length encoding based on the data contents of individual fields, which would be compromised by a block-decomposition strategy.

#### HDF5 as an initial dataset implementation

HDF5 (see http://www.hdfgroup.org/HDF5) is a data format for storing keys and their associated values in a hierarchically organised, nested collection of datasets (a column / field in ExeTera terms). HDF5 provides the ability to store large arrays of data in a column-local format (although data can be stored in multi-dimensional arrays, as is done by Pandas). It also allows for data to be stored as binary, concrete data types. HDF5 permits a user to explore the overall structure and metadata of stored datasets without loading the data itself. Data is loaded at the point that a user specifically requests the contents of a given dataset. This can be a direct fetch of the entire dataset or an iterator over the dataset. This makes it a suitable initial data format for ExeTera, although alternative columnar data storage formats are being considered to replace HDF5 for future development due primarily to issues of format fragility and shortcomings relating to concurrent reading / writing and iterator performance.

#### Field design

ExeTera supports a number of ubiquitous data types, including numeric and string formats. These are accessed in the software through various Field types. ExeTera Fields come in two basic types, dataframe-backed fields and memory fields. Any field read from a dataframe is considered dataframe-backed and is read from its dataset when requested. Memory fields are generated whenever operations are performed on fields that are not immediately written back to a dataframe. Memory backed fields can be written to dataframes. Fields have a rich API of operations that can be performed on them, including arithmetic, logical and comparison operations appropriate for their types, as well as operations such as aggregation, filtering and sorting.

##### Decoupling presentation from representation

Note that fields logically represent an array of some type of data but may have a more complex implementation involving multiple arrays that require non-trivial implementations of the operations described above. This allows ExeTera to handle string fields with highly variable lengths in an efficient manner and is only practical because each field is stored separately in the dataset.

##### Fixed string fields

Fixed string fields contain string data where each entry is guaranteed to be no longer than the length specified by the field. Fixed string fields can handle UTF8 unicode data, but this is encoded into bytes and so the specified length must consider the encoding of the string to a byte array.

##### Indexed string fields

**Fig. 4:**
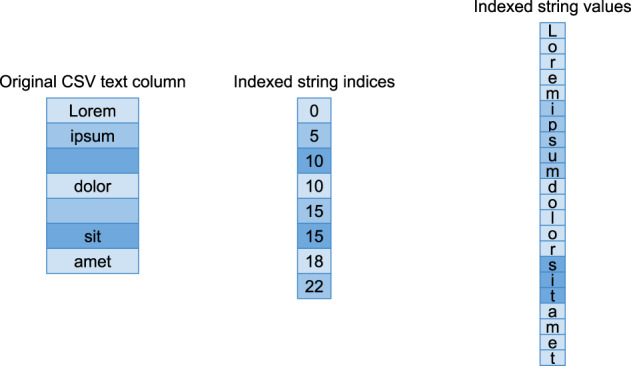
Figure illustrating how textual data is represented in ExeTera’s indexed string format. Note that empty entries are represented in the index only, as successive entries containing the same offset.

Indexed string fields are used for string data where the strings may be of highly variable length, or where a majority of string entries are empty. The data is stored as two arrays; a byte string of all of the strings concatenated together, and an array of indices indicating the offset to each entry. Figure 4 illustrates the data format for an indexed string field.

##### Numeric/logical fields

ExeTera supports the standard numeric types supported by Numpy. These are stored in a standard binary format that can be directly mapped or read as Numpy arrays.

##### Categorical fields

Categorical fields map a limited set of string values to a corresponding numeric value. A key is stored along with the field providing a mapping between string and number, e.g. 0: ‘mild’, 1: ‘moderate’, 2: ‘severe’.

##### Datetime/date fields

Datetime fields store date times as posix^26^ timestamps in double precision floating point format. The schema can also specify the generation of a ‘day’ field quantising the timestamp to the nearest day and can also specify whether the field contains empty values, in which case a filter is also generated, as with numeric fields.

#### Avoiding scaling issues with long string data

ExeTera has been explicitly designed to avoid some of the problems that Pandas (and by extension, Dask) experience when loading the Covid Symptom Study data. Pandas’ internal representation scales poorly with datasets that contain a number of string columns where one or more of the columns contain very large entries. In the case of the Covid Symptom Study Patient table, the longest string encountered in the free text data is approximately 600 characters in length. Internally, Pandas stores all string columns together in a 2D array-like structure. It allocates this array using fixed string format, where the capacity of every entry is the longest entry encountered in any of the string data. In the case of the Covid Symptom Study Patient table dated 23rd May, 2021, this means approximately 30 columns imported as string with approximately 5 million elements per column, resulting in the need to allocate a 90 GB table, despite the serialized CSV representation being only 3.8 GB. Dask uses Pandas DataFrames internally, and therefore suffers from the same degenerate memory usage. ExeTera always stores columns (fields) as distinct structures in memory, and its ability to present string data to the user through memory-efficient indexed strings means that it does not suffer from degenerate performance when dealing with datasets containing natural language fields. For the Covid Symptom Study dataset, the imported Patient table is 5.9 GB in size. Our approach further scales to enable textual analysis of natural language data on the far-larger Assessment table; 360 million assessments logged by users of the Covid Symptom Study app.

#### Data importing

Importing from CSV to strongly typed data formats requires a conversion of string data to the appropriate data type for each column in the CSV file. This is typically a one-time operation, but can be very expensive in both time and, more critically, memory. Importing can be performed in multiple stages, where the first stage imports CSV string values into string-based binary representation in the destination file format, followed by a second stage where the data is then cast to its final type. Alternatively, the data can be read and converted in a single stage. The latter is preferable but can only be performed if the data is in a clean format or appropriate converters exist to perform the operation. In the case of the Covid Symptom Study, there are a number of fields that contain values that break strongly typed constraints. Our approach in this case is to convert these columns into multiple ExeTera fields.

##### Numeric field import

Numeric data in the Covid Symptom Study is allowed to contain empty entries. These are entries where numeric data is not present. We convert these entries to a strongly typed numeric field with a user-configurable default value in place of the empty entries, and a corresponding strongly typed boolean field indicating whether data is present for that entry.

##### Categorical field import

Categorical data in the Covid Symptom Study can be a combination of expected categorical values (e.g.’mild’,’moderate’,’severe’) and free text. ExeTera provides an importer that can split this data into two fields, the categorical data itself optimally imported as numeric values with a corresponding key, and an indexed string field that can optimally store the free text data due to the typically large number of empty entries.

##### Importing via command line

Importing data from CSV can be performed via the exetera import command or via the API. The command line import command requires a schema file that describes the fields and the type conversions that they should undergo. The ExeTera schema file format is a JSON^27^ format. Each table is described by an entry inside of a JSON dictionary labelled schema. Each entry in this dictionary is the name of the table followed by the table descriptor. This has up to three entries. The first is primary_keys, which lists zero or more fields for the dataset that together represent the primary key for the table. The second is fields and contains all the field descriptors for the table. The third is foreign_keys and contains the names of foreign keys in the table and which other tables they relate to.

##### Schema file field entries

The schema file entries themselves contain at minimum a field_type entry, and depending on the specific field type, require additional entries. Box 1 shows an illustrative, minimal example of a schema file. A full specification can be found in the ExeTera github wiki listed in the *Code Availability* section.

Box 1 An illustrative, minimal example of an ExeTera JSON schema for use when importing data from CSV.

### ExeTera implementation

ExeTera is implemented in the Python programming language. Python has two aspects that make it suitable for writing software that performs data analytics and numerical analysis. Firstly, it is dynamically typed, which reduces code complexity and verbosity^28^. Secondly, it has a strong ecosystem of scientific libraries and tools to mitigate the performance and memory penalties that come with using a dynamically typed, byte-code interpreted language and runtime.

#### Use of python technologies

Code that is compiled and run directly in CPython (the reference Python implementation) executes in the Python interpreter. The Python interpreter is extremely slow relative to optimised code such as that generated by compiled, optimised C/C++; in many cases it is orders of magnitude slower (see https://numba.pydata.org/numba-doc/latest/user/5minguide.html). Python’s type system does not provide light-weight objects to represent primitive types. Even numeric values such as integers and floats are stored as full objects, and typically require 28 bytes for a 4 byte integer value. This overhead precludes efficient memory usage when iterating over large numbers of values.

Numpy^7,8^ is the Python community’s main tool for circumventing such time and space inefficiencies. Amongst other features, it provides a library for space-efficient representations of multi-dimensional arrays, and a large library of time-efficient operations that can be carried out on arrays.

The performance of such operations can be orders of magnitude faster than native CPython, but this is conditional on minimising the number of transitions between Python code and the internal compiled code in which the operations are implemented.

Not all code can be easily phrased to avoid transitions between CPython and Numpy internals. Where this is not possible, Numba^29^ is used to compile away the dynamic typing and object overhead, resulting in functions that execute at near optimised C performance levels.

### Streaming operations

Most analysis of tabular data is performed through a combination of joins, sorts, filters and aggregations. ExeTera operates on arrays of effectively unlimited length, particularly when certain preconditions are met, using the following techniques.

#### Sorting

Sorting is one of the key operations that must scale in order to process large datasets, as imposition of a sorted order enables operations such as joins to scale. ExeTera uses several techniques to provide highly scalable sorting.

##### Generation of a sorted index

ExeTera sorts data in two steps. A sorted index is first generated, represented as a permutation of the field element indices. The permuted index is then applied to each field that must be reordered.

##### Scaling multi-key sorts on long arrays

Multi-key sorts are memory intensive when keys are large, and expensive due to the internal creation of tuples in the inner loops of sorts. Multi-key sorts in ExeTera are rephrased as a series of sorts on individual keys from right to left, where the output of each sorting step is a sorted index that is the input to the next sorting step, using a stable sort. Box 2 shows pseudocode for this operation.

##### Scaling sorts on very long arrays

ExeTera has a second sorting algorithm that can be selected if an array is too large to fit into memory in its entirety. Such arrays are sorted via a two-phase approach in which the array is divided into subsets; each subset is sorted, and the sorted subsets are merged by maintaining a heap of views onto the sorted subsets. A separate index is generated and maintained with the sorted chunks, so that the merge phase is stable. Box 3 shows pseudocode for this operation.

##### Sorting multiple fields

The sorts described above, that produce a permutation of the original order, can be used to sort multiple fields in a space-efficient fashion. For large arrays, the array can be permuted in turn and written back to disk, or the permuted order maintained and reapplied when needed. ExeTera scales to provide this functionality even for very large arrays.

##### Operations on sorted fields

Many operations become merges with various predicates when performed on fields that have been sorted by the key field and can be performed in *O*(*m* + *n*) time where *m* and *n* are the lengths of the fields to be merged. This includes joins and aggregations. ExeTera performs these operations as merges when the key field is sorted. Importantly, arbitrarily large fields can be operated on in this way.

Box 2 Pseudocode for a multi-key sort that outputs a sorted index for subsequent application to many fields.

Box 3 Pseudocode for a streaming sort that outputs a sorted index for subsequent application to many fields.

**Joining**

**Generation of join maps**

Rather than performing the join on the fields themselves, ExeTera first generates primary key and foreign key index maps, which are then subsequently applied to the fields to be joined.

**Joining multiple fields**

As with sorts above, once the mapping indices have been calculated, they are applied to each field on the left side to map to the right side of the join, or vice-versa.

**Joining on sorted keys**

When the data is sorted on the keys of the respective fields, ExeTera rephrases joins as ordered merge operations.

**Aggregation**

**Generation of aggregation maps/spans**

Aggregation is another operation that ExeTera optimises through use of pregenerated indices, particularly in the case that the data is sorted by aggregation key order.

**Aggregating on sorted keys**

As with joins, when the data is sorted by the keys of the aggregated fields, aggregations are performed by ExeTera in a very scalable and efficient fashion by precomputing spans representing ranges of the key field with the same key value. This can be iterated over, and aggregations performed in a streaming fashion.

**Journalling of snapshots into a consolidated dataset**

Data for the Covid Symptom Study project is delivered as a series of timestamped snapshots. The unanonymised data generated by the Covid Symptom Study app is stored in a relational database or similar datastore, that is not accessible to query by the broader research community. Instead, the data is anonymised and then bulk exported to CSV format. The database is a live view of the dataset, however; users can update data through the app, and, unless the database is explicitly journalled and each entry made immutable, the prior states are erased. As such, a row corresponding to a given entity in two different snapshots can contain conflicting values.

**Fig. 5:**
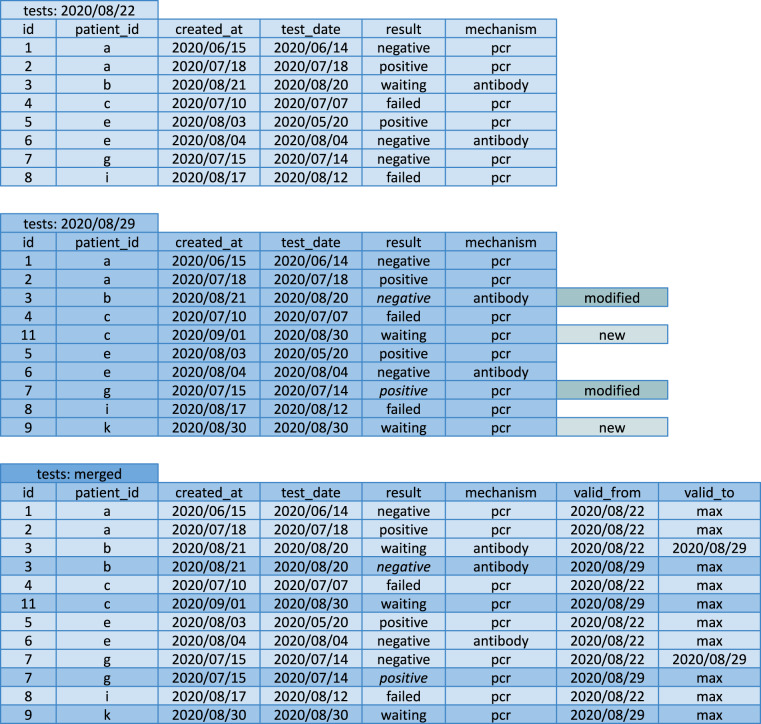
Construction of a journalled dataset. Two snapshots of a simplified dummy dataset representing COVID-19 tests; one from 2020/08/22 and one from 2020/08/29. These are used to construct a journalled dataset, bottom.

When each snapshot is large, the scaling problem is exacerbated by having to reconcile multiple snapshots. The Covid Symptom Study dataset does not have a field that reliably indicates whether the contents of a given row have changed and so determining whether a row at time *t* has changed relative to a row at time *t* + 1 requires a full comparison of all common fields. An example of this can be seen in Fig. 5.

### The ExeTeraCovid data curation pipeline

The ExeTeraCovid project provides functionality that enables a data curation pipeline incorporating data curation best practice. The pipeline has the following steps:

- Transform data from CSV to ExeTera’s HDF5 datastore format
- Perform standardised cleaning, imputation, and calculate derived values
- Run analytics pipelines on the cleaned data

The first stage is a generic operation that applies to any tabular dataset being imported into ExeTera. The second and third stages are specific to a given dataset, such as the Covid Symptom Study.

#### The covid symptom study

The Covid Symptom Study dataset is collected using the Covid Symptom Study app, developed by Zoe Ltd with input from King’s College London, the Massachusetts General Hospital, Lund University Sweden, and Uppsala University, Sweden. It is a response to the COVID-19 pandemic based on epidemiological surveillance via smartphone-based self-reporting. It asks citizens from the UK, US, and Sweden to use a mobile application to log symptoms, record COVID-19 test results and answer lifestyle and occupational questions. The Covid Symptom Study dataset has generated insights into COVID-19 that have gone on to inform government policies for handling of the disease^5,30–32^. In the UK, the App Ethics has been approved by KCL Ethics Committee REMAS ID 18210, review reference LRS-19/20-18210, and all subscribers provided consent. In Sweden, Ethics approval for the study is provided by the Central Ethics Committee (DNR 2020-01803).

As of the 23rd May, 2021, the dataset is composed of seven tables:

**Patients:** 5.08 million patients with 202 data fields. Patient records store data such as the patients’ physiological statistics, long-term illnesses, lifestyle factors, location and other data that only occasionally changes, at the patient level.

**Assessments:** 361.2 million assessments with 68 fields. Patients are asked to give regular assessments through the app that cover their current health status and symptoms, aspects of their lifestyle such as potential exposure to COVID-19, and, in early versions of the schema, any COVID-19 tests that they have had.

**Tests:** 6.98 million tests, with 20 fields. Test records are kept for each COVID-19 test that a patient has had along with the evolving status of that test (typically from’waiting’ to’positive’,’negative’, or’failed’).

**Diet:** 1.58 million diet study questionnaires with 89 fields. These ask people at several time points about their dietary and lifestyle habits.

**Vaccine doses:** 2.02 million vaccine dose entries with 14 fields. This table contains various data about the vaccine doses administered, including date, vaccine type, and vaccine course.

**Vaccine symptoms:** 8.11 million vaccine symptom entries with 35 fields. This table contains symptom data linked to the days following vaccination.

**Mental health:** 717,399 mental health entries with 56 fields. This table contains mental health survey data linked to lockdown, lifestyle habits, and general mental health.

Assessments, tests and diet study questionnaires are mapped to patients via IDs that serve as foreign keys.

This dataset is delivered as daily snapshots in CSV format. As of 23rd May 2021, the daily snapshot is 100 GB in size, and the accumulated daily snapshots are over 20 TB in size. The dataset, excepting fine-grained geolocation data, is publicly available at https://healthdatagateway.org.

#### Covid symptom study-specific cleaning and processing

The Covid Symptom Study data schema has seen rapid iteration since its inception. The initial app was rapidly released to allow users to contribute as soon as possible after the pandemic was declared and required adjustments to ensure its longevity. Furthermore, the evolving nature of the pandemic, particularly around prevalence in the population and availability and type of tests, has necessitated structural changes to the schema. Finally, this dataset is novel in terms of its scale and deployment for epidemiological analysis, and the schema has been altered to better capture data based on lessons learned during early phases of the research effort.

Public health surveillance campaigns such as the Covid Symptom Study impose time constraints on software development, with frequent changes in database structure and intense versioning to accommodate iterative refinements. The evolving epidemiology of COVID-19, the response of governments and populations to the pandemic, and academic responses to papers based on the dataset all shape the questions that are added to or removed from the app over time.

The dataset is only minimally validated at source. The fields often contain data of mixed type, and different fields can be in mutual contradiction. Numeric values are only validated for type rather than sensible value ranges. Furthermore, the dataset contains multiple competing schemas for the same underlying data, and the app version was tied to the schema version in earlier phases of the Covid Symptom Study. This resulted in users who were using older versions of the app to still contribute to otherwise retired schema elements. As such, a considerable amount of data cleaning and processing is required to extract data suitable for analysis.

##### Schema changes

The handling of COVID-19 tests in the dataset is an example of the complexity created by changes to the schema. Testing was initially reported as an assessment logging activity, but this solution had several issues. Firstly, a test needed to be logged on the day it was taken for the assessment date to be treatable as the test date. Secondly, some users interpreted the test field as something to be logged only when they took a test or received the result, whilst other users filled in intermediate assessments with the pending status. Thirdly, this system did not allow for users to enter multiple tests unambiguously. Whilst this was not a problem in the initial months of the pandemic, the ramping up of test availability necessitated a solution.

A new test table was introduced in June 2020, giving each test a unique ID to allow multiple tests for each patient. However, existing tests recorded in the old schema were not connected with new test entries, although many users re-entered old test results in the new test format. Furthermore, new tests continued to be added by users in the old, assessment-based schema format, logging on previous versions of the app. As such, there is no unambiguous way of determining whether tests in the old format are replicated by tests in the new format. This is an example of a postprocessing activity with no unambiguously correct output, which therefore requires at least a single, agreed upon algorithm to be consistently deployed to avoid inconsistencies between related analyses.

##### Validation of user-entered values (weight, height, BMI, year of birth)

In the Covid Symptom Study app, user-entered numeric values are only validated to ensure that they are numeric, as of the time of writing. There are no validations of sensible ranges given the user-selected units of measurement. Some users enter incorrect values, and some users enter values that appear sensible but only in some other unit (1.8 is a plausible height if the user is entering height in metres, for example).

##### Quality metrics for test mechanism

The Covid Test table has a ‘mechanism’ field where the user is free to either select a categorical value indicating the test mechanism, or enter free text relating to the test mechanism. Some free text clearly indicates the test type, whereas other free text entries only infer the test type weakly, through inference such as ‘home test kit’. As such, a set of gradated flags are generated that indicate the quality of the categorisation.

##### Generation of daily assessments

In case of multiple daily entries by the users, these assessments can optionally be quantised into a single daily assessment that, for symptoms, corresponds to the maximum value for each symptom that the user reported in the day. This considerably simplifies many downstream analyses.

##### Generation of patient-level assessment and test metrics

Analysis often involves the filtering of users/contributors that are categorised by aspects of the assessments and tests that they have logged. These include metrics such as whether the patient logged as being initially healthy, or whether they have ever logged a positive test result.

#### Reproducibility and algorithm immutability

Reproducibility depends on the ability to reproduce a given analysis from a version of the dataset and a set of algorithms run on the dataset. For this to be possible, algorithms must be considered immutable once implemented. This allows any subsequent version of the software to generate results consistent with those of the software version in which the algorithm was introduced.

ExeTeraCovid achieves this by requiring that a version of any given algorithm that is created is treated as immutable in the code base. This means that any target script is guaranteed to exhibit the same behaviour, provided that the following conditions hold. Firstly, any algorithms written for ExeTeraCovid are explicitly versioned. Secondly, any randomness introduced must be given consistent random seeds and, ideally, multiple sources of randomness should be given different random number generators. Once an algorithm is used in analysis, it may no longer be altered in the codebase, even if it is subsequently shown to contain errors. This enables researchers to run multiple versions of the same algorithm as part of their analytics and understand how sensitive their results are to changes and corrections. An example for this is the multiple versions of height/weight/body mass index (BMI) cleaning that have been devised over the course of the project; each is available as a separate version of the algorithm for reproducibility.

## Data Availability

The Covid Symptom Study dataset is hosted by Health Data Research UK through the https://healthdatagateway.org (HDG), by searching for “COVID-19 Symptom Tracker Dataset”. Access to the data is applied for via a two-stage process through HDG. The dataset is accessed via a protected environment provisioned by HDG for successful applicants. Access to the data is free of charge at the time of writing but HDG may in future impose cost recovery on access requests that are not related to pandemic modelling or understanding or tackling Covid-19. The code used to generate synthetic evalutation datasets is hosted at https://github.com/KCL-BMEIS/ExeTeraEval and the 10 million/100 million row synthetic dataset is available for download^33^.

## Code availability

All source code for ExeTera is made available through github under the Apache 2.0 license, at the time of writing. The code is split up into two separate projects.

**ExeTera** ExeTera is hosted at https://github.com/KCL-BMEIS/ExeTera and is available through pypi via pip install exetera.

ExeTera has a wiki that can be found at https://github.com/KCL-BMEIS/ExeTera/wiki.

**ExeTeraCovid** ExeTeraCovid is hosted at https://github.com/KCL-BMEIS/ExeTeraCovid and is available through pypi via pip install exeteracovid. Installing exeteracovid installs exetera.

ExeTeraCovid has a wiki that can be found at https://github.com/KCL-BMEIS/ExeTera/wiki.
